# Memories of *Memórias*: shaping a century of plague research and public health policy in Brazil

**DOI:** 10.1590/0074-02760250269

**Published:** 2026-05-18

**Authors:** Igor Vasconcelos Rocha, Matheus Filgueira Bezerra, Marise Sobreira, Alzira Maria Paiva de Almeida

**Affiliations:** 1 Fundação Oswaldo Cruz-Fiocruz, Instituto Aggeu Magalhães, Departamento de Microbiologia, Recife, PE, Brasil

**Keywords:** health surveillance, vector-borne diseases, yersinia pestis, plague, epidemiological studies

## Abstract

Plague, caused by *Yersinia pestis*, remains a historically significant and reemerging zoonotic threat worldwide. Often erroneously considered a medieval relic, the disease persists in natural foci, including Brazil, where it was introduced in 1899 via maritime trade.

Over the past 125 years, the country has experienced cyclical outbreaks concentrated in the northeast, where ecological conditions support enzootic transmission among wild rodents and their fleas. While improved surveillance and control have reduced human cases in recent decades, the pathogen’s zoonotic nature and potential for rapid spread in a changing climate underscore its enduring public health relevance.

*Memórias do Instituto Oswaldo Cruz* (MIOC), one of Latin America’s foremost tropical medicine journals, has been instrumental in documenting and shaping the course of plague research in Brazil. Its archives provide a unique chronological record, from pioneering studies on serum production and reservoir ecology to modern molecular analyses of bacterial virulence.

This perspective synthesises the seminal contributions published in *Memórias* that have defined our understanding of plague in Brazil, identifies critical knowledge gaps that persist, and discusses emerging challenges in an era of climate change and shifting zoonotic disease dynamics.

Plague, caused by the Gram-negative bacterium *Yersinia pestis*, represents one of history’s most devastating infectious diseases, having caused three major pandemics that shaped human civilisation.^1^ While often considered a relic of the past, plague persists as a reemerging zoonotic threat, with natural foci still active in Africa, Asia, and the Americas.^1^ ^,^ ^2^ In Brazil, plague arrived through the port of Santos in 1899, eventually establishing endemic foci in the Northeast region’s semi-arid areas, where it continues to circulate among rodent populations.^2^ ^,^ ^3^ Despite significant progress in control measures, the disease’s complex ecology, potential for rapid spread, and capacity to cause severe clinical manifestations (bubonic, septicaemic, and pneumonic forms) maintain its status as a public health concern.^1^

Over more than a century of continuous publication, *Memórias do Instituto Oswaldo Cruz* (MIOC) has served as a cornerstone for tropical disease research in Latin America,^4^ including pivotal studies on *Y. pestis* and plague epidemiology in Brazil. The journal’s archives chronicle the evolving scientific understanding of this pathogen, from pioneering serum therapy protocols^5^ to contemporary molecular investigations of bacterial virulence and transmission dynamics.^2^ ^,^ ^6^ These publications not only document Brazil’s unique experience with plague but also contribute valuable insights to the global understanding of this ancient yet persistently relevant disease.

This comprehensive perspective has three principal objectives: first, to synthesise the key contributions of MIOC to our understanding of *Y. pestis* and plague dynamics in Brazil; second, to examine current challenges in surveillance, diagnosis, and control within Brazilian foci; and third, to identify emerging research priorities at the intersection of climate change, land use transformation, and advancing genomic technologies. By systematically analysing this corpus of plague research spanning 116 years, we demonstrate how the journal has not only documented but also driven scientific progress, serving as an unparalleled resource for understanding zoonotic disease management in tropical regions.

## The birth of Fiocruz and its pivotal role in Brazil’s plague control

The dawn of the 20th century in Brazil was marked by profound public health challenges. The arrival of the bubonic plague in 1899, alongside outbreaks of yellow fever and smallpox, exposed the fragility of the nation’s sanitary infrastructure and threatened its economic hubs, particularly its bustling ports.^7^ It was within this crisis context that the Brazilian federal government initiated a campaign to modernise public health.^8^ This effort culminated in the 1900 creation of the Instituto Soroterápico Federal^9^ in the district of Manguinhos, Rio de Janeiro, under its first director, the Barão de Pedro Affonso. The institute would later be renamed Instituto Oswaldo Cruz (IOC), in recognition of Oswaldo Cruz’s role in Brazilian public health as the head of the Diretoria Geral de Saúde Pública, and become the cornerstone of the Fundação Oswaldo Cruz (Fiocruz).^10^ ^,^ ^11^

From its inception, the institute was thrust onto the front lines of the plague fight. Charged with producing immunobiologicals, the institute’s first and most urgent mission was to manufacture a vaccine and serum against *Y. pestis*.^5^ ^,^ ^10^ ^,^ ^11^ Overcoming immense technical difficulties and initial scepticism, Oswaldo Cruz and his team, which included key figures like Henrique Figueiredo de Vasconcellos, Antônio Cardoso Fontes, and Carlos Chagas,^12^ ^,^ ^13^ ^,^ ^14^ successfully established large-scale production using equine hyperimmunisation. This achievement was nothing short of revolutionary for Brazil’s public health autonomy, breaking the dependence on imported European products and ensuring a reliable supply of essential countermeasures for outbreak control.

The campaign against plague, however, extended far beyond the laboratory walls. Fiocruz scientists became instrumental in field epidemiology, investigating outbreaks and establishing the ecological foundations of the disease in the Brazilian context.^15^

The primary record of these endeavours is found in the pages of MIOC. Its early volumes are replete with original communications detailing the techniques for serum production, pathological findings from autopsies of human cases and experimental infections in animals, and meticulous descriptions of field surveys. These publications not only disseminated critical knowledge but also cemented the institute’s scientific authority, showcasing a nascent national capability to confront a pandemic threat with rigour and innovation.

The legacy of this foundational period is profound. Fiocruz’s direct involvement in plague control established a paradigm of integrating research, production, and public health intervention that defines the institution to this day.^2^ The successful containment of urban plague outbreaks in major cities like Rio de Janeiro and Santos stands as a testament to its efficacy. By generating knowledge, producing life-saving tools, and deploying expertise into the field, Fiocruz emerged as the undisputed central pillar in Brazil’s — and indeed, Latin America’s — fight against plague, a role that its flagship journal, MIOC, has documented for over a century.^4^

## From serum therapy to molecular genetics: a century of plague studies in MIOC

The extensive corpus of plague research published in MIOC provides a unique and granular perspective on the evolution of scientific thought and practice in combating this disease in Brazil. This collection of 19 pioneering articles chronicles a clear trajectory from the foundational applied studies of the “Classical Bacteriology Era” to the integrative syntheses of the modern “Contemporary Era”, all while maintaining a sharp focus on the specific epidemiological and ecological challenges of the Brazilian context (Figure). This documented evolution has produced a solid body of work instrumental in shaping the national approach to plague surveillance and control.

**Figure:**
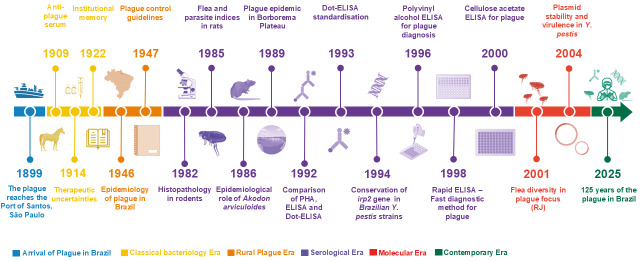
Timeline of plague research publications in *Memórias do Instituto Oswaldo Cruz*. The chart illustrates the evolution of scientific approaches to plague research in Brazil, categorised into five distinct eras based on the predominant methodological focus of the studies.

The inaugural study, published by MIOC in 1909, is a hallmark of the “Classical Bacteriology Era”, where the priorities were isolation, characterisation, and the development of specific biological countermeasures. Developed by Vasconcellos, this work established a cornerstone for plague research in Brazil by detailing an experimental protocol for anti-plague serum production in horses.^5^ The effort was quintessentially translational for its time, directly addressing the urgent public health need for locally produced biologicals and establishing Fiocruz’s role as a hub for both discovery and production.^5^

However, the pages of MIOC also reveal that the institute’s scientific function extended beyond reporting successes. Following the foundational 1909 article,^5^ subsequent studies published in the journal reflected the intense and unresolved global scientific debate about the nature of the plague bacillus’s pathogenicity. A study published in the journal exemplifies this by engaging directly with the period’s central scientific controversy.^16^ The research detailed a series of meticulous experiments that failed to isolate a free, filterable toxin from *Y. pestis* cultures using various chemical and physical methods. These results challenged the exotoxin theory of plague pathogenesis and underscored the significant difficulties in standardising an immunising antigen,^16^ thereby reflecting the profound unknowns that still surrounded the very mechanism of the disease and the therapy meant to combat it.

This foundational period of active research and debate was later curated and framed into a historical narrative by the journal itself. In 1922, the MIOC published a retrospective historical account by Vasconcellos.^17^ By dedicating its pages to this work, the journal performed a crucial act of institutional memory, consciously shaping its own legacy. The article framed the early plague studies at Manguinhos, with their methodological innovations and collective self-experimentation, not merely as past events, but as the inaugural moment of a “true bacteriological technique” in Brazil.^17^ This narrative thus served to canonise the institute’s early trajectory, a period which, from its initial laboratory triumphs to its subsequent state-sponsored scientific expeditions, is identified by broader historical analyses as a pivotal chapter for the construction of modern Brazilian science.^18^ Thus, the MIOC transformed the empirical struggles and debates it had previously documented into a coherent origin story, formally establishing the “Classical Bacteriology Era” within its own volumes as the foundational chapter in the history of Brazilian experimental science.

A significant leap in understanding the disease’s patterns occurred with two seminal epidemiological studies from the mid-20th century, reflecting the transition into the “Rural Plague Era.” This period was marked by a broadening of the public health arsenal against the disease, which came to involve integrated actions — from anti-rat campaigns and dichlorodiphenyltrichloroethane (DDT) pulverisations to the new possibilities of antimicrobial therapy. These tools began to shift the paradigm from purely immunological interventions towards a more multifaceted approach to control, particularly in non-urban settings.

The first study, published by Barreto and de Castro,^19^ systematically characterised the plague’s profile in the Northeast through a comprehensive analysis of 2,610 cases. Its key findings established that the disease predominated in areas of precarious housing (slums), confirmed the bubonic form as the most prevalent, and identified *Xenopsylla cheopis* as the dominant flea vector in tropical zones. Crucially, it quantified a stark contrast in outcomes: while the overall lethality rate was 26%, early treatment with sulphonamides drastically reduced it to 12%, underscoring the vital importance of timely intervention.^19^ Subsequently, a second study built upon this foundation to establish national control guidelines by historically evaluating measures adopted between 1936 and 1945.^20^ It reinforced the predominance of the bubonic form while highlighting the extreme severity and high lethality of the pneumonic and septicaemic manifestations. Furthermore, it provided essential evidence for the efficacy of modern insecticides like DDT in controlling flea vectors and compounds like cyanogas for rodent extermination, offering a scientific basis for transitioning to these more effective prophylactic strategies and shaping the country’s public health approach for decades.

It is relevant to note that from the late 1920s through the 1970s, much of the broader Brazilian plague research was also published in parallel journals, particularly the *Archivos de Hygiene* and the *Revista Brasileira de Malariologia e Doenças Tropicais*, reflecting the expansive and collaborative effort required to combat the disease during this period.

From the 1980s onwards, a series of pivotal investigations marked the advent of the “Serological Era”, characterised by a sophisticated use of immunology and refined field ecology to unravel the complex transmission cycles of plague.^21^ ^,^ ^22^ ^,^ ^23^ Initial research was fundamental in identifying potential wild rodent reservoirs by characterising the histopathological markers of the infection, such as hepatic necrosis and splenic atrophy, in naturally infected animals.^21^ Concurrently, complementary studies provided critical data for urban surveillance by assessing flea infestation indices, identifying seasonal peaks in *X. cheopis* prevalence that signalled periods of highest transmission risk.^22^ A significant advancement came from detailed studies on specific sigmodontine rodents,^24^ which revealed crucial ecological nuances by demonstrating that high flea infestation rates did not necessarily correlate with high susceptibility, indicating that not all abundant rodent species function as efficient reservoirs for the bacterium.

The application of these integrated techniques was proven essential during the investigation of an active outbreak in the Borborema Plateau,^25^ where bacteriological and serological diagnoses confirmed widespread *Y. pestis* circulation across 21 municipalities, with strains isolated from humans, domestic animals, and wild rodents. Further enriching this ecological understanding, a comprehensive survey in the Serra dos Órgãos Range provided the first complete inventory of the flea fauna in a distinct focus, documenting new host records and offering evidence of transmission bridges between wild and commensal rodent populations.^23^

A major and consistent thematic strand within this corpus has been the relentless refinement of diagnostic tools, marked by a focused effort to develop more sensitive, practical, and accessible serological assays. This endeavour began with a pivotal comparative study that evaluated three diagnostic tests, conclusively demonstrating the superior sensitivity of the Dot enzyme-linked immunosorbent assay (Dot-ELISA).^26^ Building on this finding, subsequent research focused on standardisation and innovation. One study meticulously optimised key parameters of the Dot-ELISA to ensure specificity and reproducibility while eliminating false-positive reactions.^27^ Simultaneously, researchers pioneered new solid phases to enhance practicality, first by creating a novel ELISA format using polyvinyl alcohol glutaraldehyde (PVA) discs, which proved highly effective for antibody detection in human samples.^28^ A significant advancement in field applicability was then achieved with the development of a rapid PVA-ELISA, which dramatically reduced the total assay time from 36 h to just 3 h without substantial loss of sensitivity.^29^ Further expanding the diagnostic toolkit, another study introduced cellulose acetate as a superior solid support. This material demonstrated enhanced performance over conventional plates and offered unique versatility by functioning effectively for both spectrophotometric reading in a standard ELISA and visual interpretation in a dot-ELISA format.^30^ Collectively, these studies epitomised the Serological Era. Through their publication, MIOC not only documented but actively disseminated this transition, moving beyond mere disease description to providing the Brazilian public health system with a robust, scalable, and practical diagnostic arsenal.

The dawn of the “Molecular Biology Era” in plague research was also captured within the journal’s pages, introducing sophisticated genetic analysis to the Brazilian context. This began with a pivotal study that surveyed the virulence-associated *irp2* gene across strains isolated from multiple outbreaks.^31^ Utilising DNA hybridisation, the research confirmed the gene’s high conservation and highlighted a significant risk in strain maintenance as the gene could be lost after *in vitro* subculture.^31^ Subsequently, a deeper investigation into genetic stability employed plasmid profiling and LD50 assays in mice to explore the relationship between plasmid content and virulence.^6^ This first comparative study of its kind across multiple strains demonstrated that plasmid loss does not always directly correlate with a reduction in virulence, challenging a long-held assumption and revealing a more complex relationship between *Y. pestis* genetics and pathogenicity.

Finally, this extensive scientific legacy was masterfully synthesised in a comprehensive review that analysed the 125-year history of plague in Brazil,^2^ representing the “Contemporary Era” of integrative science. This work meticulously documented the historical evolution of plague control, tracing the journey from the foundational efforts of the Oswaldo Cruz Era to the implementation of modern surveillance protocols. By analysing national plague service archives and historical documents, the review effectively contextualised the scientific contributions published in the journal within the broader arc of both national and global research. It underscored the enduring relevance of this rich historical data, arguing for its integration with contemporary molecular and digital technologies to build a more robust and sustainable surveillance system for the future.

The trajectory of publications in MIOC tells the story of Brazil’s scientific battle against plague. It is a narrative of evolution: from serum therapy to molecular genetics, from descriptive outbreak reports to analytical epidemiology, and from basic diagnostic methods to rapid, field-friendly assays. Each article represents a building block, contributing to a comprehensive and sophisticated understanding of *Y. pestis* that remains vital for managing this ancient threat in the modern world.

## Current challenges and knowledge gaps

Despite this formidable trajectory of scientific progress documented in MIOC, plague persists as a public health challenge in Brazil, presenting a new set of complex obstacles that defy easy solutions.^32^ ^,^ ^33^ The enzootic foci in the Northeastern, once the epicentre of human outbreaks, now presents a complex and silent threat. The primary challenge today is no longer managing large urban epidemics but understanding and monitoring the maintenance of *Y. pestis* in wild rodent populations to prevent its reemergence into human communities.^33^

A critical knowledge gap in Brazil, as in many endemic countries, is the precise understanding of the environmental and climatic drivers that govern the transition from enzootic stability to epizootic spread and subsequent spillover to humans.^33^ Globally, studies have linked plague dynamics to fluctuations in temperature, rainfall, and humidity that affect rodent and flea populations.^34^ ^,^ ^35^ ^,^ ^36^ In the Brazilian semi-arid region, characterised by extreme climatic variability and periodic droughts, the specific triggers for epizootics are not yet fully understood.^33^ ^,^ ^35^ The historical lack of long-term, systematic ecological monitoring of reservoir species and their fleas in these foci has historically hindered the development of predictive models for outbreak risk. Although initiatives have begun to address this gap, the data series remain nascent.^33^ ^,^ ^36^ ^,^ ^37^ Advancing our understanding of how environmental drivers — such as rainfall, vegetation, and altitude — interact with host and vector ecology is therefore critical for predicting the risk of new plague outbreaks in its natural foci.

Furthermore, the ecological landscape of plague in Brazil is undergoing silent but profound transformations.^32^ ^,^ ^33^ Changes in land use, agricultural expansion, and desertification are altering the habitats of known rodent reservoirs and may be facilitating the invasion of more adaptable species, potentially reshaping the transmission cycles.^32^

Another persistent challenge is the maintenance of diagnostic and surveillance capacity in the absence of human cases. The decline in reported incidents over recent decades has led to a “out of sight, out of mind” dilemma, risking a dangerous atrophy of clinical and laboratory expertise. Brazilian health professionals outside the historical foci may never encounter a plague case, leading to potential misdiagnosis and dangerous delays in treatment. This is a global issue, as seen in non-endemic countries where travel-related cases are often initially missed.

In Brazil, genomic studies have decisively answered critical questions by demonstrating that all circulating strains derive from a single introduction of the 1.ORI2 lineage during the Third Pandemic.^38^ ^,^ ^39^ The current challenge, therefore, lies not in a lack of fundamental genomic knowledge, but in its application. The imperative is to implement sustained genomic surveillance within endemic foci. This ongoing effort is crucial to rapidly distinguish between local re-emergence and new introductions, predict outbreaks by integrating genomic data with ecological and climatic variables, and monitor for emerging antimicrobial resistance.^33^ ^,^ ^39^

## Perspectives and emerging research priorities

The rich legacy of plague research published in MIOC provides not only a historical record but also a robust foundation upon which to build the next century of inquiry. The future research agenda is a direct evolution of the thematic strands so meticulously chronicled in the journal’s pages, pushing each into a new era of technological sophistication.

First, the paramount challenge remains decoding the silent maintenance of *Y. pestis* within enzootic foci. This pursuit is a direct extension of the foundational ecological studies published from the 1980s onwards, which first employed field trapping and histopathology to identify potential wild rodent reservoirs and characterise the histopathological markers of infection.^21^ ^,^ ^22^ ^,^ ^23^ ^,^ ^24^ ^,^ ^25^ The next step is to leverage this field-based approach with modern tools: employing metagenomics on soil and flea samples to identify environmental reservoirs, and integrating long-term ecological data into powerful predictive models of spillover risk. This represents the natural evolution from descriptive ecology to predictive, quantitative science.

Second, the refinement of diagnostic tools must enter its molecular phase. The emerging priority is to build upon this legacy by developing rapid, field-deployable molecular diagnostics (*e.g.*, PCR, LAMP, and CRISPR-based platforms) that can detect the pathogen itself in remote areas, in addition to antibody detection to direct, genotype-based identification. By sharing discoveries from these new frontiers, MIOC will continue to do what it has done for over a century: translate cutting-edge science into actionable knowledge. This ensures that the hard-won lessons of Brazil’s past, so vividly documented in its pages, remain central to building a more resilient and proactive defence against this ancient yet ever-evolving threat.

## Data Availability

The data supporting this study are available within the article.
